# Endogenous reprogramming of NK cell activation signal via base editing

**DOI:** 10.1016/j.omton.2026.201295

**Published:** 2026-08-01

**Authors:** Xidi Chen, Xueting Xie, Guanglei Li, Chao Yao, Cheng Fang, Shiguo Zhu

**Affiliations:** 1 Department of Immunology and Pathogenic Biology, School of Integrative Medicine, Shanghai University of Traditional Chinese Medicine, Shanghai, China; 2 Shanghai-MOST Key Laboratory of Health and Disease Genomics, NHC Key Lab of Reproduction Regulation, Shanghai Institute for Biomedical and Pharmaceutical Technologies, Shanghai, China

## Main text

Tumor-mediated immune evasion is increasingly identified as a critical barrier limiting adoptive cell therapies in solid tumors. In our recently published paper in *Cancer Research* (2026; 86: 1956–67), we address this challenge by deploying a high-fidelity base-editing platform to permanently disrupt the TIGIT inhibitory checkpoint in primary natural killer (NK) cells. This intervention leads to the complete rebalancing of receptor-ligand interactions, suggesting that reprogramming endogenous receptor networks offers a more sophisticated alternative to conventional engineering.^1^

**Figure 1:**
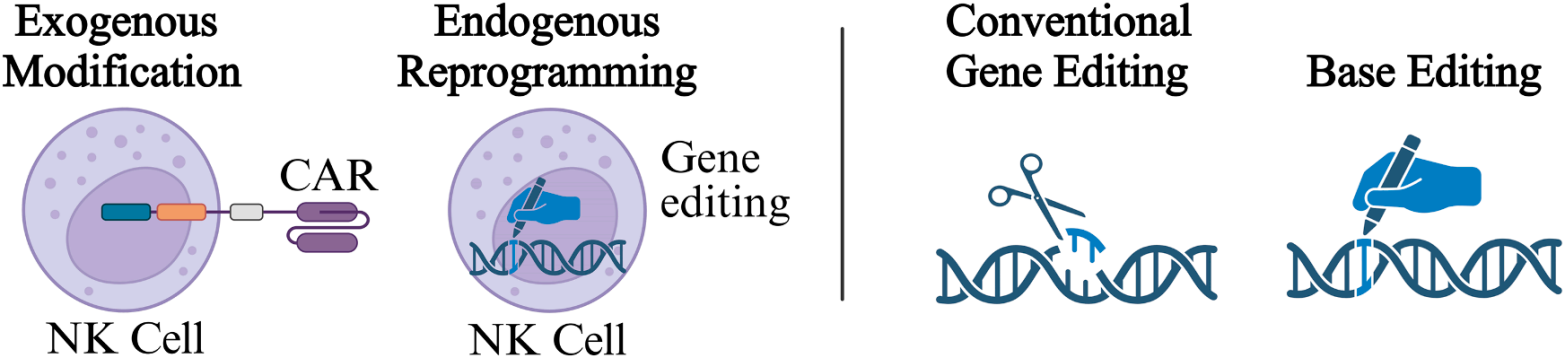
Schematic overview of NK cell engineering strategies and two major gene-editing modalities (Left) Two mainstream strategies for NK cell engineering, including exogenous CAR modification and endogenous genomic reprogramming. (Right) Mechanistic comparison between conventional double-strand break-based gene editing and break-free base editing for precise genome modification.

Fundamentally, NK cell activation relies on a fine-tuned balance of inhibitory and activating signals. To further enhance NK cell-mediated anti-tumor efficacy, we define two distinct engineering paradigms based on their strategy for signal modulation: exogenous modification and endogenous reprogramming (Figure 1). Generally, exogenous modification involves the introduction of synthetic activating receptors, such as chimeric antigen receptors (CARs), that provide additive stimulation to NK cells. Conversely, endogenous reprogramming focuses on counteracting internal inhibitory signaling through the precise ablation of immune checkpoints. By permanently eliminating these intrinsic molecular brakes, this strategy effectively lifts the inhibition and rewires the inhibitory signal into an activating one.

In general, exogenous strategies rely on integrative viral vectors, which carry genomic risks such as insertional mutagenesis, alongside the potential immunogenicity of foreign proteins. Moreover, the expression of an exogenous CAR may attenuate over time, potentially impacting long-term efficacy, or continuous tonic signaling might contribute to premature exhaustion. In contrast, endogenous reprogramming dismantles inhibitory barriers without relying on foreign integration, thereby avoiding these safety liabilities and enabling a broad spectrum of tumor recognition, and navigates the challenges by preserving the cell’s natural signaling architecture.^2^ By selectively disrupting dominant immune checkpoints, this strategy optimally harnesses cell activation without overriding the innate repertoire of activating receptors and preserves their broad-spectrum tumor recognition capabilities, allowing the therapeutic response to extend beyond a single target epitope.

It is worth noting that these paradigms are not mutually exclusive; emerging hybrid strategies increasingly combine endogenous checkpoint ablation with exogenous CAR expression or endogenous activation enhancement to achieve synergistic efficacy. Nevertheless, distinguishing their fundamental mechanisms remains crucial for optimizing safety and potency. The comparative characteristics of these two engineering paradigms, with a primary focus on endogenous checkpoint ablation, are summarized in Table 1.

**Table 1:** Comparison of exogenous modification versus endogenous reprogramming in NK cell engineering

Parameters	Exogenous modification	Endogenous reprogramming
Core strategy	Receptor grafting	Checkpoint ablation; signaling switch
Tumor recognition	Single-epitope dependent	Broad spectrum
Signaling profile	Artificial; prone to AICD/fratricide	Natural; innate repertoire
Biological fate	Subject to expression attenuation	Restored signaling architecture
Delivery vehicle	Integrative viral vectors	Non-viral RNP complex
Genomic safety	Risk of insertional mutagenesis	DSB free; mitigated off-targets
Manufacturing	High cost; logistically complex	Streamlined; scalable

NK, natural killer; AICD, activation-induced cell death; RNP, ribonucleoprotein; DSB, double-strand break.

The binding affinity between TIGIT and its ligand CD155 on tumor cells is significantly higher than that of the activating receptor CD226.^3^ Consequently, inhibitory signals naturally predominate, driving NK cells into an exhausted state.^4^ Therefore, permanent TIGIT ablation holds promise for redirecting signaling toward the activation axis. However, conventional nucleases such as CRISPR-Cas9 induce double-strand breaks (DSBs), posing risks of aneuploidy and chromosomal instability. To circumvent these limitations, base editing has emerged as a promising alternative that enables precise genetic modifications without generating DSBs (Figure 1). Table 2 summarizes the technical safety profiles of these editing modalities, highlighting the genomic preservation advantages of our base-editing approach.

**Table 2:** Comparison of conventional gene-editing and base-editing platforms

Parameters	Conventional gene editing	Base editing
Mechanism	Induces DNA DSBs	Targeted nucleotide substitution (no DSBs)
Genomic integrity	Risk of indels, aneuploidy, and translocations	High fidelity; preserves genomic integrity
Repair pathway	Relies on error-prone NHEJ/HDR	Operates independently of DSB repair
Precision	Heterogeneous indel formation via NHEJ	Precise, site-specific base conversion
p53 activation	Robust	Minimal
Applicability	Gene disruption/deletion	Single-nucleotide conversion/precision mutation

DSB, double-strand break; NHEJ, non-homologous end joining; HDR, homology-directed repair.

To safely execute this signal-conversion strategy, we utilized a Cas-embedding (CE) cytosine base editor called AccuBase, which reduces single-guide RNA (sgRNA)-independent off-target effects down to background levels.^5^ Based on the results of off-target and on-target editing efficiency, we speculate that only upon precise, sgRNA-guided binding to the target DNA does the AccuBase protein undergo conformational changes, exposing the deaminase domain outwardly to perform targeted nucleotide substitutions. To successfully manifest this structural precision in a scalable cell product, we deliver this specialized base editor directly as a pre-assembled ribonucleoprotein complex, thereby bypassing the liabilities of traditional nucleic acid vectors. This delivery strategy is particularly critical for primary lymphocytes, which are highly sensitive to foreign DNA, exposure to which frequently triggers intense cytotoxicity and accelerates cellular senescence.^6^^,^^7^ AccuBase protein overcomes the major bottleneck of difficult purification associated with conventional base-editing proteins, and good manufacturing practice (GMP)-grade material of this protein has been produced for pharmaceutical development.

Through this precise genomic ablation of *TIGIT*, we successfully rewired the immunological signaling. By redirecting tumor-derived CD155 to the activating receptor CD226, this intervention forces the tumor’s own immune evasion strategy to backfire; by eliminating TIGIT-mediated suppression, CD155 engagement shifts the balance toward CD226-driven activation, potentiating downstream ERK signaling and driving superior anti-tumor cytotoxicity. Ultimately, this molecular cascade translates the synaptic receptor switch into the potent, pan-cancer remission observed *in vivo*.

Collectively, this endogenous reprogramming strategy establishes a safe, efficient, and scalable base-editing paradigm, underscoring its potential for clinical translation. TIGIT BE-NK cells have recently secured dual Investigational New Drug (IND) clearances from China’s NMPA and the US FDA for advanced solid tumors. By transforming a dominant inhibitory checkpoint into a productive activation stimulus, this cell-intrinsic strategy offers a mechanistic alternative to conventional antibody-based checkpoint blockade. Beyond TIGIT, the same platform enables the seamless rewiring of other competitive networks, such as the HLA-E/NKG2A/NKG2C axis, thereby demonstrating a universal paradigm for converting tumor immune-evasion shields into potent activation triggers. As such, this work provides a new framework for receptor reprogramming in allogeneic cell therapy and more broadly, underscores the potential of base editing to drive the next generation of cancer immunotherapeutics.

## Acknowledgments

This study was supported by the 10.13039/501100001809National Natural Science Foundation of China (82404918, C.F.; 82373908, S.Z.). The funding agency did not play a role in the design of the study; collection, analysis, and interpretation of the data; or writing of the manuscript. We acknowledge the use of BioRender.com for the creation of graphical illustrations in this study.

## Author contributions

X.C., X.X., G.L., and C.Y. performed the experiments and wrote the original draft of the manuscript. S.Z. and C.F. conceived the study, participated in its design and coordination, and contributed to the revised version of the manuscript. All authors read and approved the final manuscript.

## Declaration of interests

S.Z. has equity and consulting relationships with Base Therapeutics. All other authors have no conflict of interest to declare.
